# Friedman Tongue Position and the Anthropometric Parameters in Adult Patients with Obstructive Sleep Apnea: An Observational Study

**DOI:** 10.3390/ijerph20043255

**Published:** 2023-02-13

**Authors:** Valeria Luzzi, Federica Altieri, Mariana Guaragna, Valentina Pirro, Beatrice Marasca, Luisa Cotticelli, Marta Mazur, Gabriele Di Carlo, Paola Di Giacomo, Carlo Di Paolo, Marco Brunori, Gaetano Ierardo, Gabriele Piperno, Giuseppe Magliulo, Annalisa Pace, Giannicola Iannella, Paolo Palange, Agnese Martini, Emma Pietrafesa, Antonella Polimeni

**Affiliations:** 1 Department of Oral and Maxillo Facial Sciences, “Sapienza” University of Rome, 00161 Rome, Italy; 2 Department of Sensory Organs, “Sapienza” University of Rome, 00161 Rome, Italy; 3 Department of Public Health and Infectious Diseases, Sapienza University of Rome, 00100 Rome, Italy; 4 Department of Occupational and Environmental Medicine, Epidemiology and Hygiene, Italian Workers’ Compensation Authority (INAIL), 00143 Rome, Italy

**Keywords:** Friedman Tongue Position, Obstructive Sleep Apnea Syndrome, sleep disorder, comorbidities

## Abstract

Introduction: Obstructive Sleep Apnea Syndrome (OSAS) is a relevant public health problem; dentists can play an important role in screening patients with sleep disorders by using validated tools and referring patients to a specialist, thereby promoting an interdisciplinary approach. The aim of the study is to identify if the OSAS severity, measured by the apnea–hypopnea index (AHI), and some anthropometric measurements are associated with the Friedman Tongue Position (FTP) within a population with dysmetabolic comorbidities. Materials and Methods: A questionnaire containing information about clinical data including height, weight, Body Mass Index (BMI), neck circumference, waist circumference, hip circumference and FTP was administered. The AHI value was measured by means of an unattended home polysomnography device. Pearson correlation coefficients were calculated, and Kruskal–Wallis, Kolmogorov–Smirnov (both nonparametric) and independence tests were performed to probe the possible relationships. The significance was set at *p* ≤ 0.05. Results: A total of 357 subjects were analyzed. The association between the FTP and AHI was not statistically significant. On the contrary, the AHI showed a positive correlation with BMI and neck circumference. A statistically significant association between the number of subjects with a larger neck and an increasing FTP class was found. BMI, neck, hip and waist circumference was associated with the FTP scale. Conclusions: although the FTP was not directly associated with OSAS severity, there was also evidence that an FTP increase is associated with an increase in the considered anthropometric parameters, and FTP can be a clinical tool used in the assessment of risk for OSAS risk factors.

## 1. Introduction

Obstructive Sleep Apnea Syndrome (OSAS) is the main breathing sleep disorder, and it is defined by the American Academy of Sleep Medicine as repeated collapses of the upper airway during sleep, resulting in a total (apnea) or partial (hypopnea) reduction in the airflow [1,2]. A pharyngeal airway which is narrow or tends to collapse constitutes the pathogenetic basis of OSAS [3]. Advanced age, alcohol use, gender, hypertension, obesity and a sedentary lifestyle are important factors in the development of OSAS [4,5,6]. The prevalence is 2–9 times higher in males than females [3,7]. In particular, the Wisconsin Sleep Cohort study stated that the prevalence of OSAS for 30–60 year old men and women is 24% and 9%, respectively [8].

It is important to improve the rates of diagnosis of OSAS due to the comorbidities and the risk of sudden death [6,9]. The main clinical signs, which are risk factors, include augmentation of the neck circumference, oropharyngeal obstruction, soft palate laxity, nasal obstructions, tonsil hypertrophy, turbinate hypertrophy, nasal septum deformity, macroglossia, retrognathia and craniofacial deformities [10,11,12]. There are several symptoms that depend on OSAS severity.

A high Body Mass Index (BMI) and body measurements such as neck circumference and waist circumference could be used as predictive factors of the presence of OSAS and its severity.

The symptoms, which include excessive daytime sleepiness, no refreshing sleep, daytime fatigue and decreased concentration, may cause impairment in social or occupational functioning [13]. In addition, OSAS can cause cardiovascular and cerebrovascular damage [14]. Polysomnography (PSG) is commonly required to diagnose sleep-related breathing disorders including OSAS.

Since dentists examine the oral cavity and have a direct view of the oropharynx, they could play an important role in screening patients with sleep disorders using validated tools and referring the patient to a specialist for final diagnosis, promoting the interdisciplinary approach. A simple tool that can be used during an intraoral examination is the Friedman Tongue Position (FTP), which could help dentists find a higher probability of sleep-disordered breathing (SDB) because it evaluates the relationship between the tongue and oral cavity.

FTP scoring is a simple, noninvasive and inexpensive technique that involves the visualization of the oropharynx. It is easy to learn and does not require any special equipment. The Mallampati system has been used for more than two decades to assess the ease of intubation in anesthesiology [15]. The American Academy of Sleep Medicine states that the Mallampati score has additional value in diagnosing OSAS in adults [16]. The modified Mallampati index (MMP), also called the Friedman Tongue Position by Friedman [17,18] and obtained without protruding the tongue, can provide a natural and physiological tongue position, similar to the one achieved during sleep.

For this reason, the primary aim of the study presented here is to evaluate the possible association between the severity of OSAS, measured with the apnea–hypopnea index (AHI), and BMI, neck circumference and the Friedman Tongue Position (FTP) score within a population with dysmetabolic comorbidities. In addition, the association between the FTP grade and the circumference of the hip and waist is investigated.

## 2. Materials and Methods

The present study was conducted at the Department of Oral and Maxillofacial Sciences of “Sapienza”, University of Rome, as part of the BRIC INAIL project SLeeP@SA [19]. The study was reviewed and approved by the Ethics Committee of Policlinico “Umberto I” (No. 6131, 18 November 2020) and was conducted in accordance with the Declaration of Helsinki.

The patients were informed in detail of the purpose of the study, and written informed consent was obtained from all participants. This project is an epidemiological study with the aim to screen OSAS in the adult working population with specific pathologies and/or conditions.

### 2.1. Study Sample

Anamnesis and clinical examinations were conducted on working patients between 18 and 65 years of age at the Department of Oral and Maxillofacial Sciences. The subjects presenting one or more of the following OSAS risk factors were asked to participate in the present study: diabetes, obesity, hypertension, heart disease and snoring. A total of 376 patients were visited in the period from January 2021 to September 2022.

### 2.2. The Questionnaire

A questionnaire divided into three sections was administered to all study participants. Each of these sections contained multiple choice and open-ended questions. The first and second sections collected personal data and information about road accidents, occupational medicine, sleep quality (e.g., the Epworth scale and Berlin questionnaire) and the type of pharmacological therapies experienced. The third section collected information about clinical data including height, weight, BMI, neck circumference, waist circumference, hip circumference, medical conditions, smoking habits, familiarity with pathologies, pathology, surgical operations, the Friedman Tongue Position (FTP) [17,18], clinical intraoral examinations and polysomnography data.

The questionnaire was a collection of questions assessing anthropometric data [10] and questionnaires (i.e., the Epworth scale and Berlin questionnaire) [2,10,20] that were already validated in the OSAS literature with the addition of information about the occupational medicine and personal habits of the subjects, which are beyond the scope of this study. As a consequence, no additional validation for the data used here was required.

### 2.3. Polysomnography

All the subjects were instructed to wear an unattended home PSG device (SOMNOtouch™ RESP eco, SOMNOmedics GmbH, Randersacker, Germany) for one or two nights. Nasal flow, pulse oximetry variations, thoracic and abdominal movements, body position information and the AHI index were recorded by the PSG device.

The AHI value was measured and considered valid if the PSG device was used at least six hours per night.

The apnea–hypopnea index (AHI), i.e., the mean number of apnea and hypoxia events that occured per hour, was used to assess the disease severity. OSAS is defined as an AHI ≥ 5 and is associated with excessive daytime sleepiness. The AASM guidelines classify OSAS severity as mild (5 < AHI ≤ 15), moderate (15 < AHI ≤ 30) and severe (AHI > 30) [21].

### 2.4. Anthropometric and FTP Data

The height and weight of the patients were measured, and the BMI of the patients was calculated as body weight (kg)/height (m^2^).

The neck circumference was measured with a standing patient at the middle of the neck, between the midcervical spine and the superior line of the cricothyroid membrane, and the waist circumference was measured at the level of the narrowest part of torso at the end of exhalation. The hip circumference was determined as the maximum value over the buttocks.

**Figure 1:**
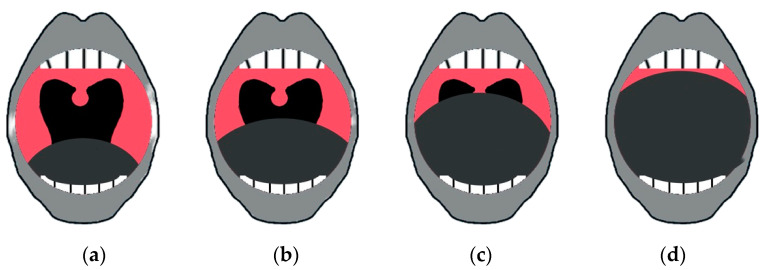
The four levels of the FTP system: 1 (**a**), 2 (**b**), 3 (**c**) and 4 (**d**).

The FTP was assessed for each patient based on the visualization of the oropharynx [18]. The patient was evaluated with their mouth open and without the protrusion of their tongue, and they were asked to open their mouth widely with their tongue left in place; the oropharyngeal crowding was graded as follows: (1) grade 1: complete visualization of the uvula, tonsils and palatal arches; (2) grade 2: the uvula, pillars and upper pole are partially visible; (3) grade 3: only part of the soft palate is visible, as the tonsils, pillars and base of the uvula could not be seen; grade 4: only the hard palate is visible (Figure 1).

### 2.5. Statistical Analysis

A descriptive analysis of the sample is given, including the percentages, means and standard deviations of the different parameters and partitions of the AHI severity classes. The Pearson correlation coefficient together with its 95% confidence interval for AHI vs BMI and AHI vs neck circumference as a function of the FTP grade were measured and the relative *p*-value is given. A linear fit was performed on the AHI vs neck circumference scatter plot, one per FTP grade, and the slope with its error is reported. A nonparametric Kruskal–Wallis test was used to assess possible associations of the AHI with the FTP, and of the FTP with neck circumference, hip circumference, waist circumference and BMI. A nonparametric Kolmogorov–Smirnov test was implemented to probe the possible difference between the neck circumference distributions of the subjects with and without OSAS. A test of independence that takes advantage of the stepping system of the FTP classes was used to study the relationship between this parameter and the neck circumference. All the test results are in the form of a *p*-value, with significance set at *p* ≤ 0.05. This study was performed both by dividing the males and females and keeping them together. The data analysis was performed using the software SPSS version 25 (IBM corporation, Armonk, NY, USA) and the ROOT framework version 6.24/04.

## 3. Results

**Figure 2:**
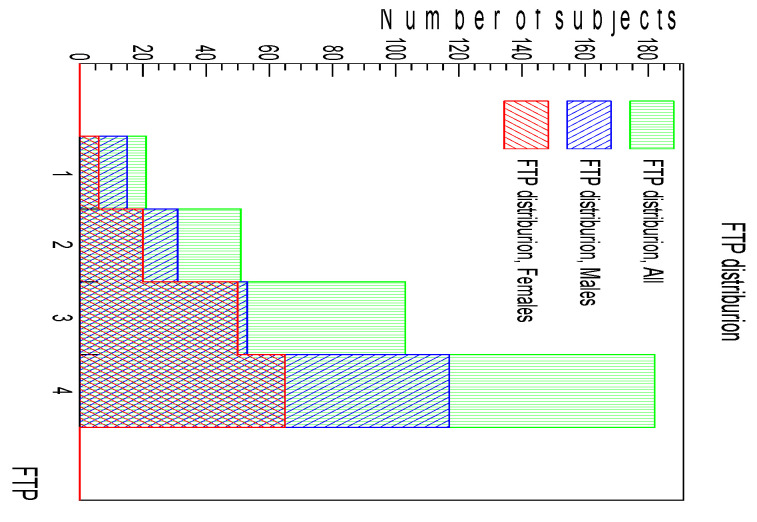
FTP distribution for all the subjects together (green) and subdivided into the male (blue) and female (red) components.

Of the 376 visited subjects, only 357 were eligible for this study: 19 subjects were excluded due to some questionnaire answer of interest missing or because the PSG result was unusable (e.g., less than 6 h of measurement or nasal cannula loss). The sample consisted of 216 males and 141 females with a mean age of 54 years with a standard deviation of 12 years. The characteristics of all 357 subjects are shown in Table 1. For a better understanding of the subdivision of the FTP classes, Figure 2 shows the numbers of subjects for each grade, for the complete sample and for the male and female components separately.

The percentages of subjects for the various inclusion criteria are as follows: diabetes, 12.3% (males 13.4%, females 10.6%); obesity, namely BMI ≥ 30 kg/m^2^, 37.8% (males 40.7%, females 33.3%); hypertension, 48.2% (males 50.5%, females 44.7%); heart disease, 8.4% (males 12.5%, females 2.1%); and snoring, 84.3% (males 89.3%, females 76.6%).

The AHI partition in the severity classes is as follows: mild, 23.8%; moderate, 22.1%; severe, 33.3%. The measured AHI for the remaining 20.7% of subjects was ≤5 events/h.

The Pearson correlation coefficient between the AHI and BMI, its 95% confidence interval and the related *p*-value were evaluated for each of the four FTP classes. These quantities are summarized in Table 2, together with the number of subjects belonging to each class. 

Apart from class 1 (where, anyhow, the 95% confidence interval was compatible with the other values), the correlation between the AHI and BMI remained moderate. In addition, the correlation value obtained considering all the subjects together was always inside the 95% confidence interval of each class.

In an analogous way, the number of subjects, the AHI vs BMI Pearson correlation coefficient, its 95% confidence interval and the *p*-value for the male and female subsamples, when divided by FTP class, are reported in Table 3.

As there were larger *p*-values, the results were less reliable, in particular for the female component. Additionally, in this case, the correlation coefficient obtained considering all the subjects belonging to the male/female subsample (last line of Table 3) was always contained within the 95% confidence interval of each individual FTP class.

Performing the same analysis with the neck circumference in place of the BMI resulted in an overall similar behavior (higher and stable correlation coefficients for classes from 2 to 4), with coefficients that were comparable or larger on average, especially for women, where it passed from 0.20 to 0.42. This was consistent with what was found in a previous study [22].

The number of subjects per each FTP grade with its AHI vs neck circumference Pearson correlation coefficient, the related 95% confidence interval and *p*-value are reported in Table 4 for the complete sample and in Table 5 for the male and female subgroups.

Given the better performances obtained using the neck circumference, a linear fit was performed on the AHI vs neck circumference scatter plot for each of the FTP grades. Only the global population was considered to obtain a more stable result. The slopes and their errors are reported in Table 4. Excluding grade 1, all the other classes showed a similar slope, indicating that the rapidity of the AHI increased as a function of the neck circumference independent of the FTP grade. This observation was also supported by the output of the Kruskal–Wallis tests performed to compare the four AHI distributions that originated when subjects were divided by their FTP class. In fact, the resulting *p*-values were p_All_ = 0.57, p_M_ = 0.66 and p_F_ = 0.22 for the complete sample and the male and female subgroups, respectively.

**Figure 3:**
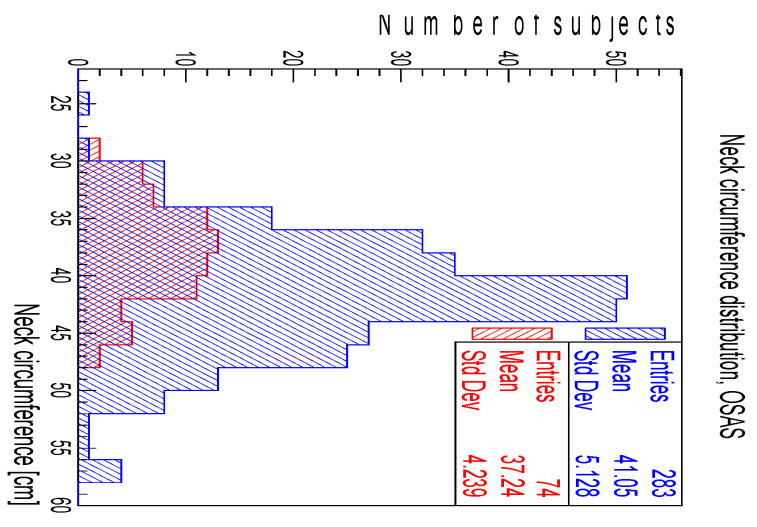
Neck circumference distribution for subjects with (blue) and without (red) OSAS.

To compare the neck circumference distributions of the subjects with and without OSAS, a Kolmogorov–Smirnov test was performed. The *p*-value of 6.8 × 10^−7^ indicated that the subjects with OSAS tended to have a larger neck, as visible in Figure 3, where the average neck circumference was ≈41 cm for the subjects with OSAS and ≈37 cm for the subjects without OSAS.

Taking into account four different anthropometric parameters, namely BMI and the circumference of the neck, hip and waist, a Kruskal–Wallis test was performed to probe if the distribution of these parameters changed as a function of the four levels of the FTP scale. The resulting *p*-values are summarized in Table 6. For the entire population, they were always <0.05, while, splitting by gender, this condition was met only in the case of neck circumference for females. These *p* < 0.05 mean that the recorded increase in the average of the corresponding parameter, when increasing the FTP grade, was statistically significant.

**Figure 4:**
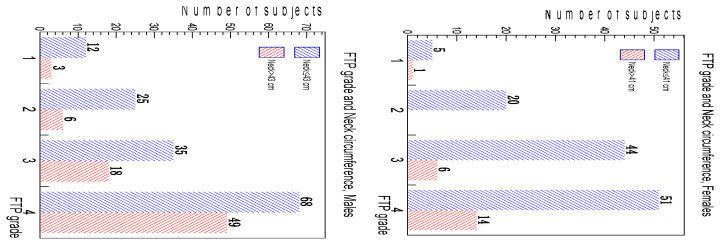
Number of subjects with a neck circumference greater than (red) or less than or equal to (blue) a threshold value for each FTP class. Threshold is set to 43 cm for males (**left**) and to 41 cm for females (**right**).

In Figure 4, the number of subjects with a neck circumference greater than (red) or less than or equal to (blue) a threshold value is shown, on the left for males (threshold = 43 cm) and on the right for females (threshold = 41 cm). A test of independence was performed on the data to investigate if the number of neck circumferences above or below the threshold depended on the FTP class. This test took into account the step structure of the FTP scale to completely exploit this characteristic: for each FTP grade, a score was needed. In this analysis, the class score was equivalent to the class level (score 1 for FTP class 1, score 2 for FTP class 2 and so on). The *p*-values were 9.4 × 10^−3^ for the males and 0.049 for the females, both indicating that the increase in the number of subjects with a larger neck when the FTP grade was increasing was statistically significant.

## 4. Discussion

The primary aim of the study presented here is to evaluate the possible association between the severity of OSAS, BMI, neck circumference and FTP score. In particular, the correlation between OSAS severity and BMI and OSAS severity and neck circumference, both as a function of the FTP scale, was measured. The sample under study consisted of subjects at high risk for OSAS [5,6,7,8,9,10,11,12], representing a possible limitation of the study itself. Excluding the first class (where the statistics were much smaller), the correlation coefficient was found to be fundamentally constant as the grade increased, suggesting that the FTP grade did not affect the strength of the linear relationship between the AHI and BMI.

Furthermore, it was observed that the AHI had a higher correlation with the neck circumference than BMI. The compatibility between linear slopes (excluding FTP class 1) indicated that the FTP grade did not modify the AHI dependence from the neck circumference and, hence, the AHI of a subject was not affected by it.

Liistro et al. [23], postulating that the combination of nasal obstruction with a high Mallampati score could increase the risk of having OSAS, found a significant association and correlation between BMI and neck circumference with AHI but an absence of association between Mallampati and AHI in subjects without nasal obstruction. The same authors stated that BMI and neck circumference are independent predictors of AHI whereas the Mallampati score is associated with AHI only in patients with nasal obstruction. These results are in agreement with and supported by the present study results, which did not show a statistically significant association between the FTP and the AHI.

In addition, the literature supports the finding that neck circumference is positively correlated with the AHI [24,25]; in particular, its correlation is higher than the BMI one, making it a better OSAS predictor. Our results agree with the literature data. A possible explanation is that the increase in fat deposition in the neck region leads to the enlargement of upper airway structures, which further leads to the narrowing and collapsing of the airway space and difficulty breathing.

Concerning the association between the FTP and anthropometric data (BMI and neck, hip and waist circumference), an increase in these parameters, as a function of an increasing FTP class, was observed. The FTP grade could provide a picture of the amount of tissue present in the posterior oropharyngeal region. The class 3 and 4 grades suggest crowding in the pharyngeal region, which makes it difficult to breathe while sleeping when the tongue collapses posteriorly [24,26].

Although the FTP was not related to the severity of the OSAS as measured by the AHI, the higher the FTP grade, the greater the BMI and circumferences average of the group.

Some studies in the literature showed that the Friedman staging system (FSS) was related positively and significantly with the severity of OSAS measured by the AHI [18,27]. In other words, patients with higher stages have a greater chance of experiencing severe OSAS. The FSS is determined by the Friedman Tongue Position (FTP), tonsil size and BMI. The present study is an epidemiological study and aimed to measure the FTP alone without using the tonsil size, which instead requires a specialist investigation. As a consequence, this could constitute a limitation of the present study. Therefore, the absence of an association between the FTP and AHI could be due to the nature of the study itself.

Considering the present study results, the use of the FTP during the clinical evaluation, although relatively quick and easy to perform, may have limited utility in predicting the severity of OSAS. Even though the FTP scoring may not be as useful among patients with a lower probability of having OSAS, it can be a useful tool when integrated with other clinical evaluations [28].

It is important for clinicians to know that OSAS is a complex disorder of multiple physiologic factors and cannot be fully described by a single physical examination finding.

The FTP, while having limitations as a diagnostic test, could be a useful part of the physical examination of patients prior to polysomnography, and it could be used to prioritize patients for polysomnography, which is an important consideration given the large backlog of patients awaiting assessment for OSAS.

## 5. Conclusions

Taking account of the present study results, conducted on a population with specific OSAS risk factors, a moderate correlation between AHI and BMI exists; moreover, neck circumference was a better indicator than BMI, being more correlated with the AHI. Subjects with OSAS had a greater neck circumference than the subjects without OSAS. Although the FTP was not directly associated with OSAS severity, there was also evidence that an FTP increase was associated with an increase in the considered anthropometric parameters and can be a clinical tool in the assessment of risk for OSAS risk factors. The FTP may still play a role when incorporated into the overall clinical picture of an overweight patient, and it still has the potential to play a role in screening for OSAS.

## Figures and Tables

**Table 1:** Subject characteristics for the complete sample and for the male and the female components.

Characteristics	All	Males	Females
	**Mean ± Std. Dev.**	**Mean ± Std. Dev.**	**Mean ± Std. Dev.**
Age (y)	54 ± 12	54 ± 11	54 ± 12
AHI (events/h)	24 ± 22	29 ± 22	17 ± 20
BMI (kg/m^2^)	29.2 ± 6.3	29.6 ± 5.8	28.6 ± 7.0
Neck circ. (cm)	40.3 ± 5.2	42.5 ± 4.5	36.8 ± 4.2
Hips circ. (cm)	112 ± 15	112 ± 12	112 ± 18
Waist circ. (cm)	103 ± 17	108 ± 15	97 ± 16
	**Median (IQR)**	**Median (IQR)**	**Median (IQR)**
FTP (grade)	4 (4–3)	4 (4–3)	3 (4–3)

**Table 2:** Pearson correlation coefficient between AHI and BMI, with its 95% confidence interval (in parenthesis) and relative *p*-value, as a function of the FTP grade. Second column reports the number of subjects per FTP class. Last line is obtained considering all the FTP classes together. * *p* < 0.05.

AHI vs. BMI—General			
FTP Grade	n. of Sub.	Corr. Coeff.	*p*-Value
1	21	0.11 [−0.30, 0.49]	0.63
2	51	0.42 [0.18, 0.62]	1.9 × 10^−3^ *
3	103	0.35 [0.17, 0.51]	2.5 × 10^−4^ *
4	182	0.37 [0.24, 0.49]	2.2 × 10^−7^ *
All	357	0.36 [0.27, 0.45]	1.5 × 10^−12^ *

**Table 3:** Pearson correlation coefficient between AHI and BMI, with its 95% confidence interval (in parenthesis) and relative *p*-value, as a function of the FTP grade for the male and female subsamples, separately. Additionally, the number of subjects per FTP class is reported. Last line is obtained considering all the FTP classes together. * *p* < 0.05.

AHI vs BMI—Division by Gender						
FTP Grade	Males			Females		
	n. of Sub.	Corr. Coeff.	*p*-Value	n. of Sub.	Corr. Coeff.	*p*-Value
1	15	0.36 [−0.13, 0.71]	0.19	6	−0.41 [−0.85, 0.35]	0.42
2	31	0.61 [0.34, 0.78]	3.1 × 10^−4^ *	20	−0.14 [−0.53, 0.28]	0.54
3	53	0.28 [0.022, 0.51]	0.040 *	50	0.37 [0.12, 0.59]	7.4 × 10^−3^ *
4	117	0.53 [0.38, 0.66]	1.0 × 10^−9^ *	65	0.13 [−0.096, 0.37]	0.25
All	216	0.47 [0.36, 0.57]	2.3 × 10^−13^ *	141	0.20 [0.039, 0.35]	0.017 *

**Table 4:** Pearson correlation coefficient between AHI and neck circumference, with its 95% confidence interval (in parenthesis) and relative *p*-value as a function of the FTP grade. Second column reports the number of subjects per FTP class, while last column reports the slope of a linear fit performed on the data. Last line is obtained considering all the FTP classes together. * *p* < 0.05.

AHI vs. Neck Circumference—General				
FTP Grade	n. of Sub.	Corr. Coeff.	*p*-Value	Slope ± Err. (Events/h/cm)
1	21	0.26 [−0.16, 0.60]	0.25	1.11 ± 0.93
2	51	0.49 [0.25, 0.67]	2.9 × 10^−4^ *	2.21 ± 0.57
3	103	0.58 [0.43, 0.69]	2.0 × 10^−10^ *	2.10 ± 0.30
4	182	0.48 [0.37, 0.59]	4.0 × 10^−12^ *	2.15 ± 0.29
All	357	0.50 [0.42, 0.57]	<10^−13^ *	−

**Table 5:** Pearson correlation coefficient between AHI and neck circumference with its 95% confidence interval (in parenthesis) and relative *p*-value as a function of the FTP grade for the male and female subsamples, separately. Additionally, the number of subjects per FTP class is reported. Last line is obtained considering all the FTP classes together. * *p* < 0.05.

AHI vs. Neck Circumference—Division by Gender						
FTP Grade	Males			Females		
	n. of Sub.	Corr. Coeff.	*p*-Value	n. of Sub.	Corr. Coeff.	*p*-Value
1	15	−0.012 [−0.47, 0.47]	0.996	6	−0.49 [−0.87, 0.26]	0.33
2	31	0.46 [0.14, 0.69]	9.4 × 10^−3^ *	20	−0.057 [−0.46, 0.36]	0.81
3	53	0.42 [0.18, 0.62]	1.7 × 10^−3^ *	50	0.47 [0.23, 0.66]	6.2 × 10^−4^ *
4	117	0.50 [0.35, 0.62]	1.3 × 10^−8^ *	65	0.45 [0.24, 0.62]	1.7 × 10^−4^ *
All	216	0.45 [0.34, 0.55]	4.7 × 10^−12^ *	141	0.42 [0.27, 0.55]	2.3 × 10^−7^ *

**Table 6:** *p*-values from Kruskal–Wallis tests performed between the indicated anthropometric parameter (BMI and circumference of neck, hip and waist) and the FTP. * *p* < 0.05.

Parameter vs. FTP	All	Males	Females
BMI	0.0077 *	0.15	0.079
Neck circ.	0.010 *	0.23	0.025 *
Hip circ.	0.041 *	0.20	0.19
Waist circ.	0.014 *	0.21	0.15

## Data Availability

The data presented in this study are available upon request from the corresponding author due to restrictions.
